# Editorial: Current trends on emotional processing: contributions of acquired brain injury, neurodegenerative diseases, and ageing

**DOI:** 10.3389/fpsyg.2024.1378270

**Published:** 2024-02-29

**Authors:** Beatriz García-Rodríguez, Álvaro Ruiz-García, Carolina Peña-Martínez

**Affiliations:** ^1^Basic Psychology-II Department, National University of Distance Education, Madrid, Spain; ^2^Hospital Universitario 12 de Octubre, Madrid, Spain; ^3^Department of Psychology, Faculty of Health Sciences, Rey Juan Carlos University, Madrid, Spain; ^4^Health Neuroscience Institute, Byrd Alzheimer's Center, University of South Florida, Tampa, FL, United States

**Keywords:** emotions, emotion processing, emotion regulation, emotion impairments, traumatic brain injury, aging

Emotions play a crucial role in people's social and adaptive lives. Two key emotional skills are emotional processing and emotional regulation, both of which depend, in part, on the integrity of brain functioning and age. In this topic, we aim to summarize current work exploring the affective life of people with acquired brain injury (ABI) and how age may modulate their emotional processes. The interest in these studies is 2-fold: theoretical, providing evidence regarding the type of emotional processing (automatic or controlled), and applied, enabling the design of intervention strategies to improve the affective life of individuals with ABI.

Emotional processing involves understanding the emotional meaning of any stimulus. From a methodological perspective, many researchers have considered emotional facial expression as the most universal and, therefore, the most suitable stimulus to investigate questions associated with the type of processing (e.g., Ekman, 1993; García-Rodríguez et al., 2012). In this monograph, we present two articles investigating the relationships between affective and cognitive processes and their possible interdependence.

Gray et al. examined the accuracy in emotional processing of basic emotions and the impact of both the cognitive deficit associated with age and depressive disorders on this processing. Their aim was to determine whether understanding emotions requiring a greater cognitive load declines with age and whether this decline is more pronounced in patients with depression. Results showed the fulfillment of the positivity phenomenon: joy processing remains intact over the years, while negative emotions, particularly fear, seem to be affected, attributed to the greater difficulty in cognitively processing these negative emotional stimuli. Regarding depressive disorders, no significant differences were found, suggesting that deficits in understanding emotional expressions may not be due to depression. An association with non-emotional cognitive deficits was explored, revealing that only disgust was associated with a lack of cognitive resources, opening new lines of research into the processing of discrete basic emotions.

In a similar vein, Turkstra et al. present a study where ABI patients and non-damaged individuals identify basic emotions presented in two modalities: only the face and the face in its context, allowing participants to designate the emotion reflected in each face. Results indicated that the ABI group used fewer terms to describe each emotion than the control group. Eye movements were measured to rule out attentional deficits in ABI patients, revealing no differences between the two groups. The authors propose a theoretical explanation for this unexpected result, suggesting that ABI patients exhibit a certain emotional apathy that makes them less involved in emotional tasks.

These two articles address a relevant issue in affective neuroscience, presenting a novel methodology with results that again question whether emotional processing is genuinely controlled and, therefore, dependent on cognitive capacity integrity.

Another crucial aspect of understanding emotions is the ability of individuals to regulate their emotional response. Emotional regulation implies, to some extent, an individual's control over their response, providing insights into the type of processing. In situations where the emotional response is closely related to risk, the response would be automatic, with little or no control whatsoever. Conversely, in social situations, individuals without brain damage can exercise relative control over their emotional responses.

In a parallel line of research, Challakere Ramaswamy, Butler, Ton, Wilhelm, Mitchell, Knight, Greenberg, Ellis, Allnutt et al. studied the emotional responses of people with ABI, specifically the possible association between brain damage and impulsivity, aggressiveness, and violence. From a large sample of almost 700 ex-offender participants, their contributions highlight that 55% present brain damage, aligning with data found in the psychological literature (Shiroma et al., 2010; Farrer and Hedges, 2011; Durand et al., 2017). Furthermore, 55% of brain-damaged participants exhibit higher rates of impulsive and violent emotional expression on questionnaires of anger, irritability, impulsivity, and anger expression, with this difference increasing according to the degree of brain damage.

The orbitofrontal cortex (OFC) is one brain area responsible for regulating behavior, but it also plays a role in olfactory processing. Challakere Ramaswamy, Butler, Ton, Wilhelm, Mitchell, Knight, Greenberg, Ellis, Gebski et al. measured the olfactory identification ability of ex-offenders with the Sniffin-sticks test. Results are noteworthy: 16% of participants could be classified as hyposmic. Considering these results, the authors conclude that olfactory functioning can serve as an indicator of orbitofrontal functioning, opening a new line of research regarding the use of olfactory tests as predictors of emotional dysregulation.

In conclusion, this monograph comprehensively addresses the influence of emotions on the lives of people with ABI and the modulation of these processes by age. The reviewed studies propose novel methodologies that question the nature of emotional processing, raising inquiries about its cognitive control. Additionally, the emotional responses of people with ABI are explored in relation to impulsivity, aggressiveness, and violence, revealing a significant association with brain damage. The relationship between olfactory functioning and emotional regulation, particularly through the orbitofrontal cortex, is presented as a relevant finding, suggesting the potential use of olfactory tests as indicators of emotional dysregulation. Together, these results provide new perspectives for psychological intervention and suggest future lines of research in the field of affective neuroscience and the understanding of emotional expressions in people with ABI.

## Author contributions

BG-R: Conceptualization, Supervision, Writing – original draft, Writing – review & editing. ÁR-G: Supervision, Writing – original draft, Writing – review & editing. CP-M: Writing – review & editing.
